# Pyrimidine-4-carb­oxy­lic acid

**DOI:** 10.1107/S1600536813012610

**Published:** 2013-05-15

**Authors:** Katarzyna Kiegiel, Wojciech Starosta, Janusz Leciejewicz

**Affiliations:** a Institute of Nuclear Chemistry and Technology, ul. Dorodna 16, 03-195 Warszawa, Poland

## Abstract

The crystal structure of the title compound, C_5_H_4_N_2_O_2_, is built of acid mol­ecules located on a mirror plane. They form sheets stacked along the *b-*axis direction. The mol­ecules inter­act *via* O—H⋯N hydrogen bonds, forming [001] chains, and weak van der Waals inter­actions.

## Related literature

For the structure of a Li complex with pyrimidine-4-carboxyl­ate and aqua ligands, see: Starosta & Leciejewicz (2012 ▶).
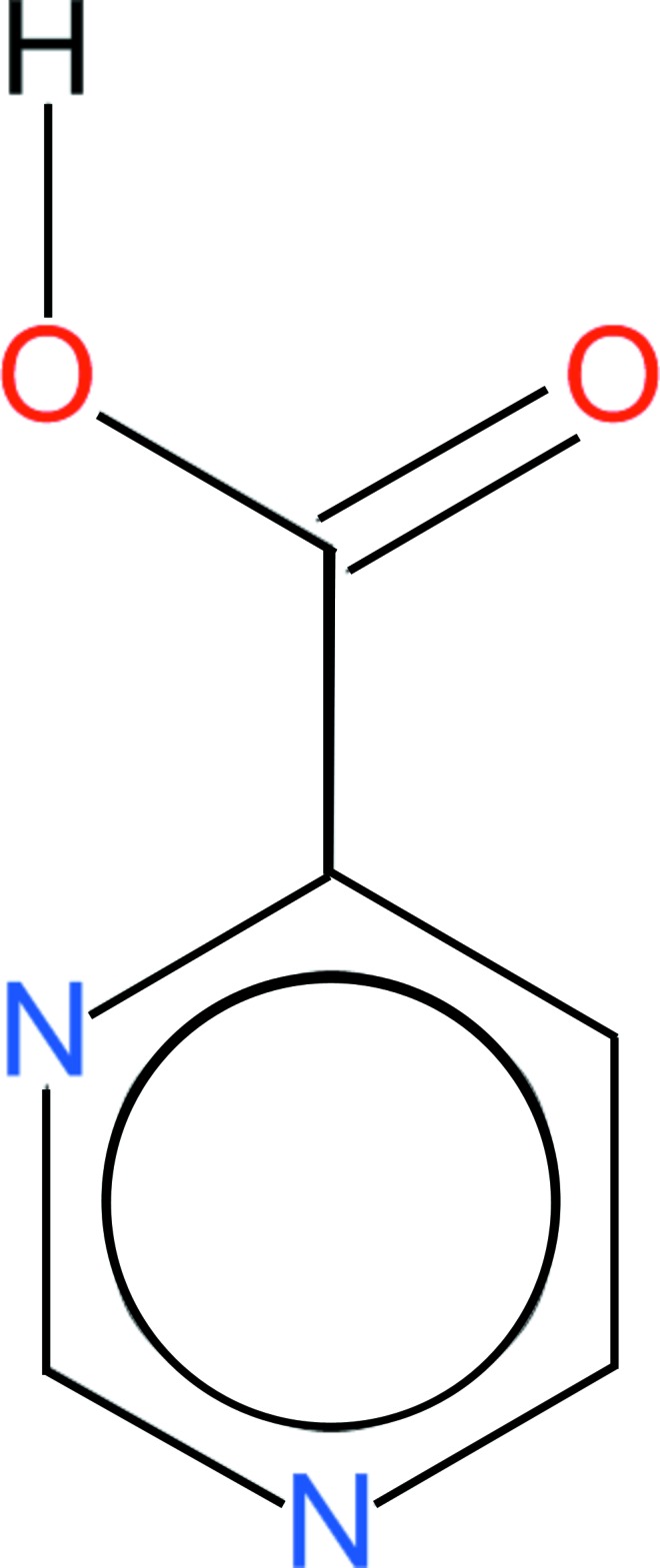


## Experimental

### 

#### Crystal data

C_5_H_4_N_2_O_2_
*M* *_r_* = 124.10
Monoclinic, 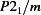

*a* = 6.0080 (12) Å
*b* = 6.3519 (13) Å
*c* = 7.4834 (15) Å
β = 112.20 (3)°
*V* = 264.41 (9) Å^3^
*Z* = 2
Mo *K*α radiation
μ = 0.12 mm^−1^
*T* = 293 K
0.17 × 0.16 × 0.06 mm

#### Data collection

Kuma KM-4 four-circle diffractometer
Absorption correction: analytical (*CrysAlis RED*; Oxford Diffraction, 2008 ▶) *T*_min_ = 0.973, *T*_max_ = 0.994
1981 measured reflections
545 independent reflections
349 reflections with *I* > 2σ(*I*)
*R*_int_ = 0.129
3 standard reflections every 200 reflections intensity decay: 0.9%

#### Refinement

*R*[*F* ^2^ > 2σ(*F* ^2^)] = 0.048
*wR*(*F* ^2^) = 0.124
*S* = 1.00
545 reflections
58 parameters
H atoms treated by a mixture of independent and constrained refinement
Δρ_max_ = 0.15 e Å^−3^
Δρ_min_ = −0.34 e Å^−3^

### 

Data collection: *KM-4 Software* (Kuma, 1996 ▶); cell refinement: *KM-4 Software*; data reduction: *DATAPROC* (Kuma, 2001 ▶); program(s) used to solve structure: *SHELXS97* (Sheldrick, 2008 ▶); program(s) used to refine structure: *SHELXL97* (Sheldrick, 2008 ▶); molecular graphics: *SHELXTL* (Sheldrick, 2008 ▶); software used to prepare material for publication: *SHELXTL*.

## Supplementary Material

Crystal structure: contains datablock(s) I, global. DOI: 10.1107/S1600536813012610/bt6903sup1.cif

Structure factors: contains datablock(s) I. DOI: 10.1107/S1600536813012610/bt6903Isup2.hkl

Supplementary material file. DOI: 10.1107/S1600536813012610/bt6903Isup3.cml

Additional supplementary materials:  crystallographic information; 3D view; checkCIF report

## Figures and Tables

**Table 1:** Hydrogen-bond geometry (Å, °)

*D*—H⋯*A*	*D*—H	H⋯*A*	*D*⋯*A*	*D*—H⋯*A*
O1—H1⋯N1^i^	1.04 (4)	1.62 (4)	2.660 (3)	179 (3)

Symmetry code: (i) 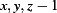.

**Fig. 1.**
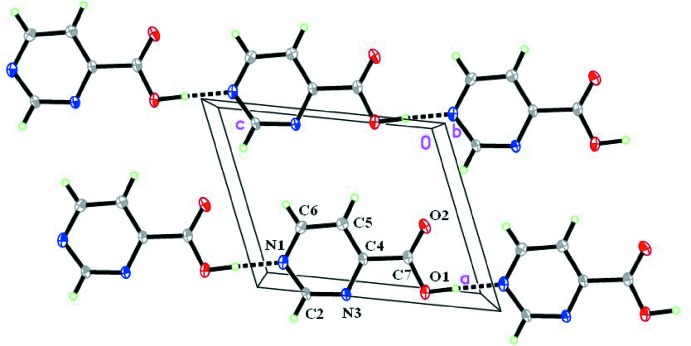
Molecules of the title compound with atom labelling scheme and 50% probability displacement ellipsoids viewed along the b axis.

## supplementary crystallographic information

### Comment

The monoclinic structure of pyrimidine-4-carboxylic acid C_5_H_4_N_2_O_2_ is composed of molecular sheets stacked along [010] crystal direction (Fig.1). Within a sheet, hetero-ring and carboxylate group atoms are coplanar. Acid molecules interact *via* hydrogen bonds of 2.658 (3) A in which protonated carboxylate O atoms are as donors and hetero-ring N atoms in adjacent acid molecules act as acceptors. The C—C and C—N bond distances and bond angles within the acid molecule do not differ from those reported earlier in the structure of the Li complex with the title acid (Starosta & Leciejewicz, 2012). The sheets are held together by van der Waals interactions as indicated by the distance between adjacent sheets which is 3.171 (1) A.

### Experimental

75 ml of a hot (*ca* 350 K) aqueous solution containing 31.6 mmol of potassium permanganate was added dropwise during 3 h to 8 ml of stirred aqueous solution containing 21.3 mmol of 4-methylpyrimidine and 5 mmol of NaOH. After stirring for half an hour longer, 1 ml of methanol was added to decompose the excess of potassium permanganate. The hot solution was filtered and the solid washed twice with 5 ml of water. Then, the filtrate and the washings were concentrated to *ca* 15 ml and acidified to pH 2–3 with concentrated HCl. After cooling to room temperature the precipitate containing 10.5 mmol of crude pyrimidine-4-carboxylic acid was recrystalized from a mixture of water and methanol taken in 20:1 ratio to give 1.1 g. (8.9 mmol) of colourless crystal blocks of the title acid (yield 42%, m.p. 508–509 K).

### Refinement

The hydrogen atom attached to the carboxylic group was located in a difference map and refined isotropically, while the three H atoms attached to pyrimidine C atoms were located at a calculated positions and treated as riding on the parent atoms with C—H=0.93 Å.

### Crystal data

**Table:**

C_5_H_4_N_2_O_2_	*F*(000) = 128
*M**_r_* = 124.10	*D*_x_ = 1.559 Mg m^−^^3^
Monoclinic, *P*2_1_/*m*	Mo *K*α radiation, λ = 0.71073 Å
Hall symbol: -P 2yb	Cell parameters from 25 reflections
*a* = 6.0080 (12) Å	θ = 6–15°
*b* = 6.3519 (13) Å	µ = 0.12 mm^−^^1^
*c* = 7.4834 (15) Å	*T* = 293 K
β = 112.20 (3)°	Plate, colourless
*V* = 264.41 (9) Å^3^	0.17 × 0.16 × 0.06 mm
*Z* = 2	

### Data collection

**Table:**

Kuma KM-4 four-circle diffractometer	349 reflections with *I* > 2σ(*I*)
Radiation source: fine-focus sealed tube	*R*_int_ = 0.129
Graphite monochromator	θ_max_ = 25.7°, θ_min_ = 2.9°
profile data from ω/2θ scan	*h* = −7→7
Absorption correction: analytical (*CrysAlis RED*; Oxford Diffraction, 2008)	*k* = −7→7
*T*_min_ = 0.973, *T*_max_ = 0.994	*l* = −9→9
1981 measured reflections	3 standard reflections every 200 reflections
545 independent reflections	intensity decay: 0.9%

### Refinement

**Table:**

Refinement on *F*^2^	Primary atom site location: structure-invariant direct methods
Least-squares matrix: full	Secondary atom site location: difference Fourier map
*R*[*F*^2^ > 2σ(*F*^2^)] = 0.048	Hydrogen site location: inferred from neighbouring sites
*wR*(*F*^2^) = 0.124	H atoms treated by a mixture of independent and constrained refinement
*S* = 1.00	*w* = 1/[σ^2^(*F*_o_^2^) + (0.0689*P*)^2^] where *P* = (*F*_o_^2^ + 2*F*_c_^2^)/3
545 reflections	(Δ/σ)_max_ < 0.001
58 parameters	Δρ_max_ = 0.15 e Å^−^^3^
0 restraints	Δρ_min_ = −0.34 e Å^−^^3^

### Special details

**Table:**

Geometry. All e.s.d.'s (except the e.s.d. in the dihedral angle between two l.s. planes) are estimated using the full covariance matrix. The cell e.s.d.'s are taken into account individually in the estimation of e.s.d.'s in distances, angles and torsion angles; correlations between e.s.d.'s in cell parameters are only used when they are defined by crystal symmetry. An approximate (isotropic) treatment of cell e.s.d.'s is used for estimating e.s.d.'s involving l.s. planes.
Refinement. Refinement of *F*^2^ against ALL reflections. The weighted *R*-factor *wR* and goodness of fit *S* are based on *F*^2^, conventional *R*-factors *R* are based on *F*, with *F* set to zero for negative *F*^2^. The threshold expression of *F*^2^ > σ(*F*^2^) is used only for calculating *R*-factors(gt) *etc*. and is not relevant to the choice of reflections for refinement. *R*-factors based on *F*^2^ are statistically about twice as large as those based on *F*, and *R*- factors based on ALL data will be even larger.

### Fractional atomic coordinates and isotropic or equivalent isotropic displacement parameters (Å^2^)

**Table:**

	*x*	*y*	*z*	*U*_iso_*/*U*_eq_	
O1	1.0036 (4)	0.2500	0.2735 (3)	0.0416 (6)	
C4	0.8398 (5)	0.2500	0.5171 (4)	0.0308 (7)	
N1	0.9072 (4)	0.2500	0.8961 (3)	0.0397 (7)	
N3	1.0647 (4)	0.2500	0.6476 (3)	0.0371 (7)	
O2	0.6049 (4)	0.2500	0.1835 (3)	0.0649 (8)	
C7	0.8036 (5)	0.2500	0.3063 (4)	0.0364 (7)	
C5	0.6416 (5)	0.2500	0.5672 (4)	0.0403 (8)	
H5	0.4860	0.2500	0.4747	0.048*	
C6	0.6868 (5)	0.2500	0.7631 (4)	0.0448 (9)	
H6	0.5573	0.2500	0.8024	0.054*	
C2	1.0847 (5)	0.2500	0.8311 (4)	0.0401 (8)	
H4	1.2401	0.2500	0.9240	0.048*	
H1	0.967 (6)	0.2500	0.126 (5)	0.064 (11)*	

### Atomic displacement parameters (Å^2^)

**Table:**

	*U*^11^	*U*^22^	*U*^33^	*U*^12^	*U*^13^	*U*^23^
O1	0.0403 (11)	0.0677 (13)	0.0196 (9)	0.000	0.0145 (8)	0.000
C4	0.0308 (14)	0.0389 (14)	0.0225 (13)	0.000	0.0097 (11)	0.000
N1	0.0401 (13)	0.0606 (16)	0.0208 (11)	0.000	0.0142 (11)	0.000
N3	0.0276 (11)	0.0651 (16)	0.0173 (12)	0.000	0.0071 (9)	0.000
O2	0.0364 (12)	0.130 (2)	0.0206 (10)	0.000	0.0020 (9)	0.000
C7	0.0366 (15)	0.0507 (16)	0.0217 (13)	0.000	0.0109 (12)	0.000
C5	0.0287 (14)	0.0613 (19)	0.0293 (15)	0.000	0.0093 (13)	0.000
C6	0.0325 (14)	0.072 (2)	0.0342 (15)	0.000	0.0179 (12)	0.000
C2	0.0304 (13)	0.0718 (19)	0.0160 (12)	0.000	0.0062 (11)	0.000

### Geometric parameters (Å, º)

**Table:**

O1—C7	1.314 (4)	N3—C2	1.332 (3)
O1—H1	1.04 (4)	O2—C7	1.200 (3)
C4—N3	1.335 (3)	C5—C6	1.386 (4)
C4—C5	1.376 (4)	C5—H5	0.9300
C4—C7	1.509 (3)	C6—H6	0.9300
N1—C6	1.322 (4)	C2—H4	0.9300
N1—C2	1.329 (4)		
			
C7—O1—H1	110.9 (19)	C4—C5—C6	116.4 (3)
N3—C4—C5	122.8 (2)	C4—C5—H5	121.8
N3—C4—C7	118.1 (2)	C6—C5—H5	121.8
C5—C4—C7	119.1 (3)	N1—C6—C5	122.4 (3)
C6—N1—C2	116.0 (2)	N1—C6—H6	118.8
C2—N3—C4	115.2 (2)	C5—C6—H6	118.8
O2—C7—O1	124.9 (2)	N1—C2—N3	127.3 (2)
O2—C7—C4	120.6 (3)	N1—C2—H4	116.4
O1—C7—C4	114.5 (2)	N3—C2—H4	116.4

### Hydrogen-bond geometry (Å, º)

**Table:**

*D*—H···*A*	*D*—H	H···*A*	*D*···*A*	*D*—H···*A*
O1—H1···N1^i^	1.04 (4)	1.62 (4)	2.660 (3)	179 (3)

Symmetry code: (i) *x*, *y*, *z*−1.
